# Draft Genome Sequence of *Aeromonas hydrophila* Strain Ae25, Isolated from a Septicemic Moribund Koi Carp (*Cyprinus carpio*) in Sri Lanka

**DOI:** 10.1128/genomeA.01523-17

**Published:** 2018-02-01

**Authors:** Karim Honein, S. S. S. De S. Jagoda, A. Arulkanthan, Hideki Ushio, Shuichi Asakawa

**Affiliations:** a Laboratory of Marine Biochemistry, Department of Aquatic Bioscience, Graduate School of Agricultural and Life Sciences, the University of Tokyo, Yayoi, Bunkyo-ku, Tokyo, Japan; b Laboratory of Aquatic Molecular Biology and Biotechnology, Department of Aquatic Bioscience, Graduate School of Agricultural and Life Sciences, the University of Tokyo, Yayoi, Bunkyo-ku, Tokyo, Japan; c Center for Aquatic Animal Disease Diagnosis and Research, Department of Veterinary Pathobiology, Faculty of Veterinary Medicine and Animal Science, University of Peradeniya, Peradeniya, Sri Lanka

## Abstract

Motile aeromonad septicemia caused by mesophilic strains of *Aeromonas hydrophila* is a widespread problem in cultured freshwater fish. We announce here the draft genome sequence of the multidrug-resistant *A. hydrophila* strain Ae25, isolated from a koi carp (*Cyprinus carpio*) with motile aeromonad septicemia that was collected from an ornamental fish-breeding farm in Sri Lanka.

## GENOME ANNOUNCEMENT

*Aeromonas hydrophila* is a rod-shaped Gram-negative bacterium found in freshwater habitats (1, 2). It is an opportunistic pathogen associated with severe diseases and infections in both fish and humans. In cultured freshwater fish, it is implicated in hemorrhagic septicemia (3). *A. hydrophila* has also been isolated from fish, meat, shrimps, mussels, and milk destined for human consumption (4), highlighting the public health risk. In addition, the global increase in fish aquaculture and an excessive and often unregulated use of antimicrobial drugs for the treatment of fish infections have led to the surge in antibiotic-resistant strains (5). *Aeromonas* species, such as *A. hydrophila*, are emerging human pathogens with a strong ability to acquire resistance to antibiotics and facility of transfer to humans via pets and food fish (6).

The present strain was isolated from the kidney of a moribund koi carp (*Cyprinus carpio*) showing signs of septicemia that was collected from an ornamental fish-breeding farm in Sri Lanka (1). Genomic DNA was extracted from a pure culture of the isolate (1) using the DNeasy blood and tissue kit (Qiagen), and a genomic DNA library was constructed with the Ion Xpress Plus fragment library kit (Life Technologies, Inc.). DNA was sequenced on the Ion Torrent PGM platform (Life Technologies, Inc.) using the Ion 318 Chip with 400-bp single-read chemistry.

Reads were examined with FastQC (7) and quality trimmed using FASTX-Toolkit (http://hannonlab.cshl.edu/fastx_toolkit/index.html). Reads with a minimum of 75% bases of Q20 or over were selected, resulting in a total of 1,050,018 reads (303,493,887 bp). Reads were first assembled into contigs using SPAdes version 3.5.0 (8); then, the contigs were further manually joined using CLC Genomics Workbench version 8 by alignment against reference genomes (GenBank accession numbers CP000462 and CP005966). The assembled genomes were assessed using QUAST version 3.0 (9).

The assembly of *A. hydrophila* strain Ae25 resulted in 45 contigs >300 bp (largest contig, 444,768 bp; *N*_50_, 248,050 bp), with a total length of 4,761,654 bp and an average G+C content of 61.25%. Both the size of the genome and its G+C content are in agreement with the data of published *A. hydrophila* genomes (10, 11).

Annotation of the genome of Ae25 with Rapid Annotations using Subsystems Technology (RAST) (12) identified 4,764 coding sequences (CDSs). In particular, RAST predicted 108 genes related to virulence, disease, and defense. Further, RAST identified 25 genes encoding multiple resistance efflux pumps, 3 genes for β-lactamase, 1 gene for the multiple antibiotic resistance (MAR) locus, 1 gene for lysozyme inhibitors, and 4 genes encoding resistance to fluoroquinolones. Ten genes involved in the regulation of virulence were also recognized.

Further, 69 tRNAs and 8 rRNAs were predicted using tRNAscan-SE 2.0 (13) and RNAmmer 1.2 (14), respectively. The phage search tool PHAST (15) detected two intact prophages showing identities to phiO18P, an *Aeromonas* bacteriophage (GenBank accession number NC_009542) (16) and YMC11/07/P54_PAE_BP, a *Pseudomonas* phage (GenBank accession number NC_030909).

The draft genome sequence of *A. hydrophila* Ae25 will help us comprehend the genomic features and intraspecific variations that define the pathogenicity of this opportunistic organism.

### Accession number(s).

The draft genome sequence of *A. hydrophila* Ae25 has been deposited in DDBJ/EMBL/GenBank under the accession numbers BEYT01000001 to BEYT01000045.
